# In Vitro Assessment of *Penicillium expansum* Sensitivity to Difenoconazole

**DOI:** 10.3390/microorganisms12112169

**Published:** 2024-10-28

**Authors:** Mohammed Khadiri, Hassan Boubaker, Abdelaaziz Farhaoui, Said Ezrari, Mohammed Radi, Rachid Ezzouggari, Fouad Mokrini, Essaid Ait Barka, Rachid Lahlali

**Affiliations:** 1 Phytopathology Unit, Department of Plant Protection, Ecole Nationale d’Agriculture de Meknès, Km10, Rte Haj Kaddour, BP S/40, Meknes 50001, Morocco; khadiri.eps@gmail.com (M.K.); farhaoui.aziz22@gmail.com (A.F.); simodefes13@gmail.com (M.R.); ezzouggarirachid@gmail.com (R.E.); 2 Laboratory of Microbial Biotechnologies and Plant Protection, Faculty of Sciences, Ibn Zhor University, BP 8106, Agadir 8000, Morocco; h.boubaker@uiz.ac.ma; 3 Laboratory of Biotechnology and Valorization of Bio-Resources (BioVaR), Department of Biology, Faculty of Sciences, Moulay Ismail University, BP 11201, Zitoune, Meknes 50000, Morocco; 4 Microbiology Unit, Laboratory of Bioresources, Biotechnology, Ethnopharmacology and Health, Faculty of Medicine and Pharmacy Oujda, University Mohammed Premier, Oujda 60000, Morocco; saidez@live.fr; 5 Laboratory of Environment and Valorization of Microbial and Plant Resources, Faculty of Sciences, Moulay Ismail University, BP 11201, Zitoune, Meknes 50000, Morocco; 6 Laboratory of Biotechnology, Conservation and Valorisation of Natural Resources (LBCVNR), Faculty of Sciences Dhar El Mehraz, Sidi Mohamed Ben Abdallah University, Fez 30000, Morocco; 7 Biotechnology Unit, Nematology Laboratory, Regional Center of Agricultural Research of Rabat, National Institute of Agricultural Research, Avenue Ennasr, BP 415 Rabat Principale, Rabat 10090, Morocco; fmokrini.inra@gmail.com; 8 Induced Resistance and Plant Biosection Research Unit, EA 4707-USC INRAE1488, Reims Champagne-Ardenne University, 51687 Reims, France

**Keywords:** *P. expansum*, apple, sensitivity, difenoconazole, resistance, EC50

## Abstract

*Penicillium expansum* causes blue mold, a major post-harvest disease affecting apples. This disease is commonly managed using fungicides, including Difenoconazole (Dif), a demethylation inhibitor (DMI) approved for its control. This investigation aims to evaluate the baseline sensitivity of 100 *P. expansum* isolates to Difenoconazole. The isolates were collected from symptomatic apples in 34 storage warehouses across the Fes-Meknes and Draa-Tafilalet regions over three years (2020, 2021, and 2022). The study revealed an increase in the percentage of inhibition of mycelial growth and spore germination of *P. expansum* proportional to the increasing concentration of the fungicide. Moreover, the results indicate that 46 isolates were able to develop even at a concentration of 5 µg/mL of Dif (the suggested discriminatory dose), indicating reduced sensitivity to this fungicide. The analysis of the values of the effective concentration to inhibit 50% (EC50) of mycelial growth of *P. expansum* ranging from 0.027 to 1.673 µg/mL (mean: 0.263 µg/mL, variation factor: 62.507) and for spore germination from 0.0002 to 0.787 µg/mL (mean: 0.048 µg/mL, variation factor: 4113.835). The wide variation in EC50 values indicates significant variability in the isolates’ responses to Dif, likely due to diverse sampling in space and time. Our results showed that some *P. expansum* isolates could grow even at high concentrations of Dif, indicating limited efficacy of this treatment. The EC50 of five isolates exceeded 0.92 µg/mL, suggesting potential resistance. This study indicates reduced sensitivity and possible emergence of resistant strains. Notably, it is the first evaluation of *P. expansum* sensitivity to Dif in Morocco.

## 1. Introduction

*P. expansum* is a member of the ascomycetes that cause blue rot, a severe post-harvest disease of apples and pears worldwide [1]. *P. expansum* is considered the main post-harvest pathogen of pome fruits, damaging apples and other deciduous fruits in the field, during harvesting, packaging, or storage [2]. A study of post-harvest diseases in Washington State showed that blue rot accounted for 28% of fruit rots in warehouses. As a result, blue mold causes between 50 and 250 million US dollars’ worth of damage every year [3]. This disease is represented by a soft, watery, slightly brown rot that is also characterized by the appearance of blue-green conidia covering the surface of the fruit, which manifests itself in the advanced stages of the rot [4].

The presence of pathogenic fungi on fruit can considerably elevate the risk of causing serious illness in human consumers. *P. expansum* causes blue rot in fruit while producing toxic secondary metabolites such as patulin and citrinin [5]. Patulin, a cyclic tetraketide, has been shown to have a highly toxic impact on both plant and animal cells, due to its interactions with the cellular sulfhydryl groups essential for proteins, as well as with glutathione. Patulin has been linked to a range of adverse effects, including mutagenic risks, genotoxic consequences, actions hurting the immune system, teratogenic potential, and neurotoxic results [6]. The same toxic effects can be attributed to citrinin, in particular, it is associated with nephrotoxic effects, immunotoxic effects, and potential teratogenic effects, making this mycotoxin potentially harmful to human health [7].

Fungicides have been widely used to combat blue rot and other post-harvest diseases. Worldwide, post-harvest treatment against *P. expansum* primarily relies on thiabendazole (Figure S1), which belongs to the benzimidazole family, and imazalil, an inhibitor of sterol demethylation (DMI). These substances are typically applied through pre-storage dipping or as an in-line treatment during the packaging process as part of the effort to combat post-harvest diseases in apples [8]. In 2004, the USA also registered two new fungicides for pome fruit: fludioxonil (FDL) and pyrimethanil (PYR). Both are effective against blue rot [9].

In 2016, difenoconazole (Figure S1), a new fungicidal demethylation inhibitor (DMI) molecule, was registered for post-harvest use on pome fruits. This product forms part of the mixture with FDL and is marketed under the name AcademyTM by Syngenta Crop Protection [1]. Furthermore, Jurick et al. [10] reported that difenoconazole exhibited both curative and protective activities, effectively controlling *Penicillium* spp., which is responsible for blue mold in stored apples. Difenoconazole (DIF) has a systematic action as well as an important ability to control fungal infections, as recently illustrated [1]. DMIs, like DIF, attack sterol 14a-demethylase Cytochrome P450 (CYP51), an essential component of fungal membrane sterols required for proper membrane function [11].

*P. expansum* is a fungus with a significant risk of fungicide resistance. As a result, resistance against TBZ, associated with various mutations in the b-tubulin gene, is reported worldwide in many production regions [12]. The presence of PYR resistance has recently been noted in the USA in north-western regions, but it is still not significant. Recently, low levels of resistance to FDL or reductions in susceptibility have appeared sporadically in a few apple packinghouses [1]. For fungicides belonging methylation inhibitor (DMI) family, a recent study of laboratory mutants of *P. expansum* resistant to the DMI difenoconazole revealed that resistance was linked to a mutation in the PeCYP51 gene [13].

In a previous study, an assessment of apple storage conditions in Moroccan facilities was conducted, along with a sampling of rotten apples [14]. This study revealed that post-harvest treatments were primarily based on three active ingredients: thiophanate-methyl, carbendazim, and difenoconazole. However, given that the first two substances have been withdrawn from the market, it is crucial to evaluate the sensitivity of pathogens to difenoconazole [14]. Furthermore, the study also reported that 72% of apple rots were caused by *Penicillium expansum*, emphasizing the need to assess the response of this pathogen to the chemical treatment, considering the increasing resistance of pathogens to certain active substances [13]. In this context, the present study aims to evaluate the sensitivity of 100 *Penicillium expansum* isolates, obtained from rotten apples collected from various storage facilities in Morocco.

## 2. Materials and Methods

### 2.1. Sampling Locations and Fungal Isolation

**Figure 1:**
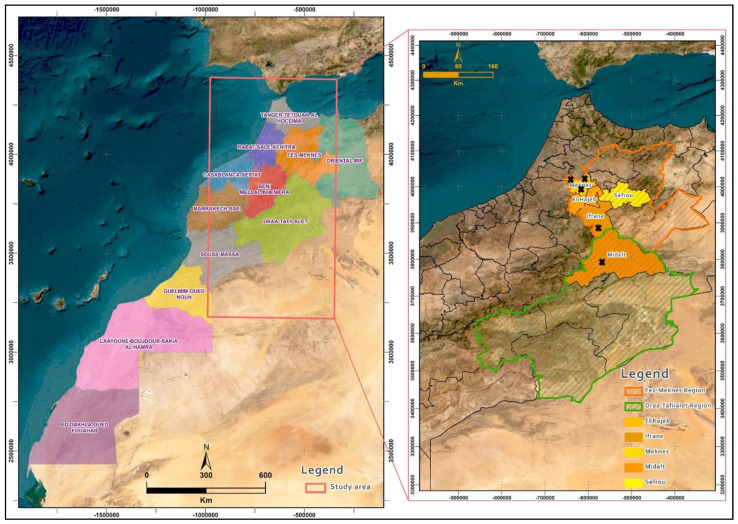
Sampling areas in the Fes-Meknes and Draa-Tafilalet regions of Morocco. This map was generated using ArcGIS Pro version 2.6. The black crosses indicate the locations of *P. expansum* isolates resistant to difenoconazole.

Rotten apples from various cultivars were collected from 34 storage warehouses in the Fes-Meknes (Meknes, El Hajeb, Sefrou, and Ifrane) and Draa-Tafilalet (Midelt) regions of Morocco over a period of 3 years, namely, 2020, 2021, and 2022 (Figure 1, Table 1). These apples are characterized by a brown mold with distinct edges, developing in cushion-like patches on the surface, first white and then blue-green (Figure S2). All samples were placed in sterile plastic bags and transported to the Laboratory of Phytopathology at the National School of Agriculture in Meknes. The process of isolating fungal pathogens involved disinfecting symptomatic samples with a 2% sodium hypochlorite solution, followed by two rinses with sterile distilled water. Subsequently, the samples were air-dried under a laminar flow hood. Using a sterile scalpel, three pieces were carefully excised from the margin of decay on each apple. These pieces were placed in Petri dishes containing Potato-Dextrose-Agar (PDA) culture medium. The dishes were then incubated at 25 °C for seven days in the dark using an IN 30 cultivator (Memmert GmbH Co., Cologne, Germany). Several subcultures on PDA medium were performed to obtain pure isolates [15,16]. In this study, 100 isolates were identified as *Penicillium expansum* using appropriate identification keys [17,18,19]. The isolates obtained were stored at 4 °C until use.

### 2.2. Molecular Identification of Fungal Isolates

To confirm the morphological identification of the fungal isolates, molecular identification was also conducted. Genomic DNA extraction followed the method outlined by Doyle and Doyle [20]. Approximately one square centimeter of each sample was placed in an extraction tube with 500 µL of extraction buffer. The mixture was crushed with a pestle, vortexed, and incubated at 65 °C for 30 min with intermittent rocking. After incubation, the samples were centrifuged at 13,000 rpm for 5 min, and 400 µL of the supernatant was mixed with 400 µL of chloroform/isoamyl alcohol (24:1). This mixture was gently agitated for 5 min and centrifuged again at 14,000 rpm for 5 min. Then, 350 µL of the supernatant was precipitated with an equal volume of isopropanol, mixed by rocking and centrifuged at 14,000 rpm for 10 min. The supernatant was discarded, and the pellet was washed with 500 µL of 70% ethanol, vortexed, and centrifuged for 5 min at 14,000 rpm. The pellet was dried at 60 °C for 30 to 45 min and resuspended in 50 µL of sterile distilled water (SDW). The extracted DNA was stored at −20 °C. DNA quantification and quality were assessed using a NanoDrop spectrophotometer (Jenway Genova Nano, Serial No 67281, Cole-Parmer, Stone, Staffordshire, UK). Polymerase chain reaction (PCR) was performed using specific primers of *Penicillium expansum*: PatF (GenBank Accession No. AIG62137): patF-F (ATGAAATCCTCCCTGTGGGTTAGT, Eurogentec 7412543) and patF-R (GAAGGATAATTTCCGGGGTAGTCATT, Eurogentec 7412544), A final volume of 25 μL was used for each PCR reaction. The reaction mix included 5 µL of PCR buffer (5×), 1 µL (10 µM) of each primer, 0.2 µL (5 U/µL) of EnzimaGoTaq DNA polymerase (Bioline, London, UK), 15.3 µL of SDW and 2.5 µL of genomic DNA. For the negative control, genomic DNA was replaced by SDW. The PCR was carried out using a thermocycler according to the following conditions: an initial denaturation at 94 °C for 5 min, followed by 40 cycles of amplification, each cycle of which consisted of denaturation at 94 °C for 45 s, primer annealing at 65 °C for 45 s and extension at 72 °C for 30 s and the last cycle was followed by a final extension for 10 min at 72 °C [21]. PCR products were visualized on a 1.5% agarose gel (Bioline: Agarose, Molecular Grade, Meridian Bioscience, Memphis, Tennessee, TN, USA) using a UV transilluminator (Quantum CX5 Edge—Gel Documentation System, France) to assess the presence and size of amplicons following electrophoresis. The electrophoresis was conducted using a Tris-Borate EDTA (TBE) buffer (0.5×), prepared by dissolving 5.39 g of Tris (Sigma Life Science, Alexandria, VA, USA), 2.75 g of boric acid (Fisher Scientific International Company, Waltham, MA, USA), and 0.29 g of EDTA (Polysciences, INC., Warrington, PA, USA) in 1 L of distilled water [14].

### 2.3. Fungicide

The commercial fungicide formulated with Difenoconazole (Score 250EC, Syngenta, L1042661 MOR/05W-PPE 4095375), distributed by Syngenta Morocco and manufactured by Syngenta Plant Protection S.A. Basel, Switzerland, was used to assess the sensitivity of 100 isolates of *P. expansum* to this active substance belonging to the Demethylation Inhibitors (DMI) family.

### 2.4. Sensitivity Assay of Mycelial Growth to Difenoconazole

One hundred isolates of *P. expansum* collected from different apple storage warehouses were tested for the sensitivity of their mycelial growth to Difenoconazole (Dif). Indeed, concentrations 0.00, 0.01, 0.5, 1, 5, and 10 µg/mL of Dif were obtained by adding appropriate quantities of fungicide to an autoclaved PDA medium cooled to approximately 50 °C. The concentration of 5 µg/mL was considered the discriminatory dose [10]. The inoculum was prepared by transferring spores from a 7-day-old PDA culture of each isolate to a tube containing 1 mL of sterile distilled water with 0.01% Tween 20. The concentration was adjusted to 1 × 10^6^ spores/mL using a hematocytometer. The spore suspension was poured into PDA medium and the cultures were incubated at 25 °C for 24 h. Afterward, mycelial plugs of 5 mm in diameter were cut out and placed in the center of the PDA plates modified with the different concentrations of Dif. PDA plates without fungicide were used as a control [9,22]. Four Petri dishes were used for each isolate. Colony diameter was measured after 10 days at 25 °C. The experiment was performed twice. Then, for each isolate, percentage inhibition of mycelial growth PI (MG) was calculated according to the following formula: PI (MG) = (Dc − Df)/Dc × 100; with Dc representing the average diameter of the fungal colonies in the control (medium without fungicide) and Df corresponding to the average diameter of the fungal colonies in the medium amended with the fungicide for all concentrations examined [23]. The effective concentration that reduced mycelial growth to half (EC50) was calculated from the regression equation (y = ax + b) between the percentage inhibition and the log10 of the fungicide concentration [24].

### 2.5. Spore Germination Assay

Spore suspensions were prepared in tubes containing potato dextrose broth (PDB) medium using the same method described previously. This PDB medium containing *P. expansum* spores was amended with Dif to obtain final concentrations of 0.00, 0.01, 0.5, 1, 5, and 10 µg/mL. The tubes without fungicide were used as a control. Four repetitions per isolate were made. After 24 h of incubation at 25 °C, germination inhibition of 100 spores in each isolate was examined for each dose using a light microscope (at a magnification of 10× 40×). A spore was considered germinated if the length of the germ tube was equal to or greater than the diameter of the spore. The percentage inhibition of spore germination PI (GI) was calculated as follows: PI (GI) = (Gc − Gf/Gc) × 100; where Gc and Gf represent respectively the average number of spores germinated in the control and the medium amended with the fungicide [25,26,27]. This experiment was conducted twice. The effective concentration to inhibit 50% of spore germination (EC50) were calculated in the same manner described in the previous section.

### 2.6. Statistical Analyses

EC50 values were calculated for each isolate based on the regression equation between percentage inhibition and Log10 of fungicide concentration. All data were subjected to statistical analysis of variance (ANOVA) using SPSS software (version 25, IBM SPSS Statistics 25). Separation of means was carried out using the Tukey test with a significance level of *p* < 0.05.

## 3. Results

### 3.1. Molecular Identification of Penicillium expansum Strains

**Figure 2:**
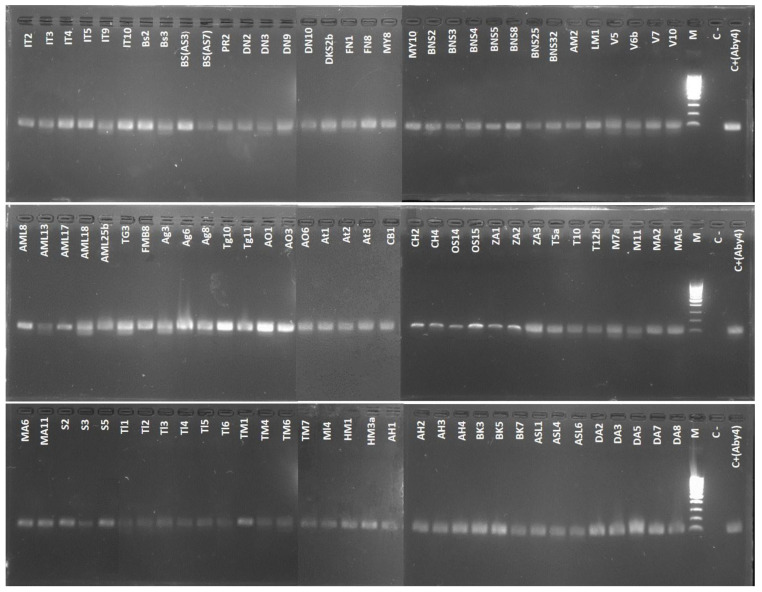
Agarose gel electrophoresis profiles (1.5%) showing PCR-amplified products with *P. expansum*-specific primers; patF-F and patF-R of the patF gene. Lane M: GeneRuler 100 bp DNA Ladder (Thermo Scientific). Lane C-: negative control. Lane C+ (Aby4): positive control with the Aby4 strain of *P. expensum* identified through molecular sequencing of the internal transcribed spacer (ITS) region of rDNA (accession number: OR426630).

PCR conducted with the patF gene-specific primers confirmed that all 100 fungal isolates analyzed belong to the species *Penicillium expansum*. These isolates were collected from symptomatic apples in 34 storage facilities located within five Provincial Agriculture Directorates (PAD) in the main apple-producing regions of Morocco, indicating significant geographical diversity. The positive control used in this study, Aby4, had previously been identified as *P. expansum* through molecular sequencing of the ITS region of rDNA (accession number OR426630), ensuring the reliability of the results obtained with the molecular marker patF (Figure 2).

### 3.2. Effect of Difenoconazole on Mycelial Growth of P. expansum

**Figure 3:**
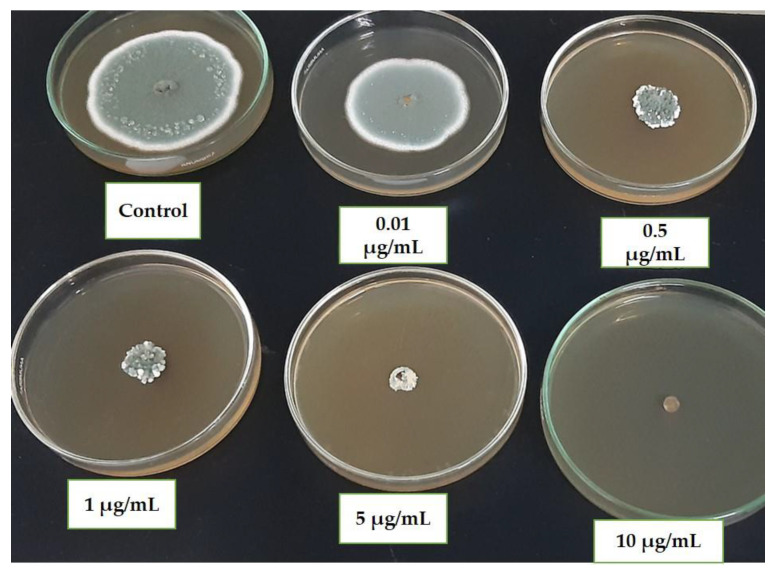
Effect of different difenoconazole concentrations on Mycelial Growth of *P. expansum*.

Analysis of variance revealed a significant effect of fungicide concentration on the percentage of mycelial growth inhibition among the 100 isolates tested (Table S1). The inhibition percentage increased with higher concentrations of difenoconazole, with complete inhibition observed for some isolates at a concentration of 5 µg/mL (the suggested discriminatory dose). Additionally, the results indicate that 46 isolates (out of the 100 studied) were able to grow even at 5 µg/mL of difenoconazole (Table 2, Figure 3). These *P. expansum* isolates were sampled from symptomatic apples collected from various storage stations across the five PADs of this investigation: Meknès, El Hajeb, Sefrou, Midelt, and Ifrane. In contrast, a concentration of 10 µg/mL inhibited the growth of all *P. expansum* isolates (Table 2, Figure 3).

### 3.3. Effect of Difenoconazole on Spore Germination of P. expansum

Statistical analysis of the results revealed a significant difference in spore germination inhibition percentages among the different *P. expansum* isolates (Table S1), with inhibition increasing proportionally to the rising fungicide concentration. Total inhibition was observed at a concentration of 0.5 µg/mL for four isolates: IT2 (Meknes), MA6 (Ifrane), At2 (Sefrou), and AH1 (Midelt). In contrast, at this same concentration, no complete inhibition of mycelial growth was observed for any isolate. Additionally, 1 µg/mL of difenoconazole prevented the germination of 56 *P. expansum* isolates. However, only nine isolates were able to germinate at a concentration of 5 µg/mL, specifically: M7a from Ifrane, Tl2 and Tl4 from Midelt, ZA2 and ZA3 from Sefrou, DN3 and BNS5 from Meknes, and AML13 and AML25b from El Hajeb. Among these nine isolates, four are the same whose mycelium was able to grow at this concentration (among the 46), however the remaining five are different. None of the isolates was able to germinate at a concentration of 10 µg/mL (Table 2). These findings indicate that *P. expansum*’s response to difenoconazole varies between developmental stages, with spore germination showing significantly higher sensitivity to the fungicide than mycelial growth.

### 3.4. Analysis of EC50 Values

The EC50 values provide insights into the concentration of the fungicide needed to inhibit half of the mycelial growth and spore germination in 100 isolates of *P. expansum*. The results obtained showed a wide variation in the EC50 values for all the isolates studied. For the mycelial growth, the EC50 values ranged from 0.027 to 1.673 µg/mL, with a mean of 0.263 µg/mL and a variation factor of 62.507. In the spore germination test, the EC50 values spanned from 0.0002 to 0.787 µg/mL, with a mean of 0.048 µg/mL significantly lower than that of mycelial growth and a notably high variation factor of 4113.835. These findings indicate that the isolates in our study exhibit greater variability in sensitivity to difenoconazole (higher VF), with both lower and higher EC50 values compared to those reported in the reference study. Notably, our results reveal significantly lower average EC50 values for spore germination inhibition, with some isolates showing much greater sensitivity, as evidenced by the minimum value. However, the high VF reflects a broad range of responses, with certain isolates demonstrating substantial resistance (Table 3).

**Figure 4:**
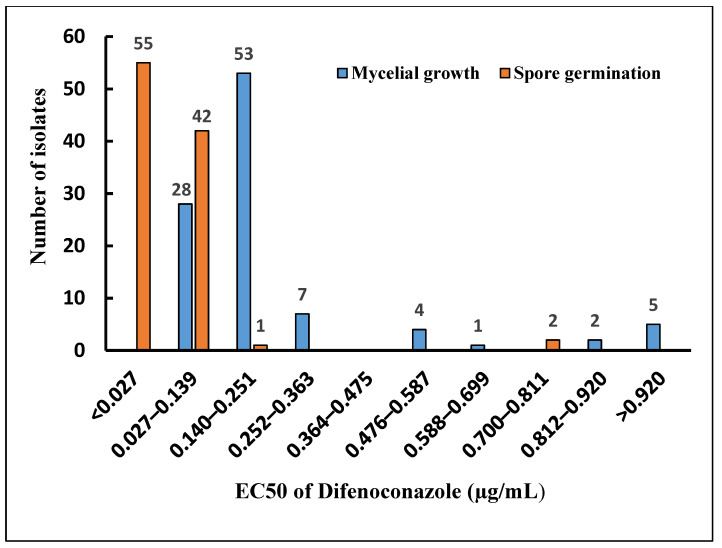
Distribution of EC50 values for mycelial growth and spore germination of 100 isolates of *P. expansum*.

The results depicted in Figure 4 illustrate the distribution of EC50 values for mycelial growth and spore germination of *P. expansum* across different concentration intervals of Dif. The majority of isolates demonstrated EC50 values ranging from 0.027 to 0.139 µg/mL (28 isolates) and from 0.140 to 0.251 µg/mL (53 isolates) for mycelial growth. Conversely, 55 isolates displayed an EC50 below 0.027 µg/mL for spore germination, representing the minimum EC50 value for mycelial growth, while the EC50 of 42 isolates was between 0.027 and 0.139 µg/mL, indicating a high sensitivity of *P. expansum* spore germination to low concentrations of Dif. It is noteworthy that in the highest concentration intervals, five isolates demonstrated an effective concentration to inhibit half of the mycelial growth greater than 0.920 µg/mL. According to a reference study [1] these five *P. expansum* isolates exhibit resistance to difenoconazole. The EC50 values of the remaining isolates were distributed across the other intervals. These findings highlighted that almost all *P. expansum* isolates (98%) showed a very high sensitivity to Dif for spore germination, compared to mycelial growth, which exhibited remarkable variability with a tolerance to the fungicide even at high concentrations.

The five *P. expansum* isolates resistant to difenoconazole (Bs (AS3), Bs (AS7), DN9, M11, and DA7) are geographically distributed across the study regions (Figure 1). Specifically, three of these isolates are located in storage facilities under the Provincial Agriculture Directorate (PAD) of Meknes, one isolate in the PAD of Ifrane, and another in the PAD of Midelt. This distribution indicates that the majority of resistant isolates are concentrated in the storage facilities belonging to the PAD of Meknes.

## 4. Discussion

*P. expansum*, the causal agent of blue mold in post-harvest apples, is the most dominant pathogen among other fungal agents affecting apples in Morocco (72%), with an extremely high pathogenicity. It causes significant economic losses in the pomiculture sector. To control this storage disease, the chemical approach based on synthetic fungicides remains the most widely adopted in most Moroccan storage facilities, with Dif, registered under the commercial name Score, being the most commonly used currently [14]. However, the frequent use of this active substance could lead to pathogen resistance to Dif. Due to the significant lack of research regarding the evaluation of the efficacy of this fungicide in Morocco; this study holds considerable importance as it aims to assess the sensitivity of *P. expansum* to Dif.

The results obtained demonstrated a significant effect of Dif concentration on the inhibition percentage of mycelial growth in the 100 isolates tested. As the Dif concentration increased, so did the inhibition percentage, with complete inhibition beginning at a concentration of 5 µg/mL, which was suggested as a discriminatory dose. Interestingly, even at a concentration of 5 µg/mL, 46 isolates of *P. expansum* were capable of growth. However, the concentration of 10 µg/mL completely inhibited the growth of all *P. expansum* isolates. In this regard, the study performed by Jurick et al. [10] revealed that complete inhibition of mycelial growth, except for three isolates, occurred at 5 µg/mL Dif, while 10 µg/mL did not support growth for any of the isolates examined. The authors of that investigation recommended a concentration of 5 µg/mL Dif for phenotyping *Penicillium* spp. isolates with reduced sensitivity. In comparison with this research, our findings indicated that nearly half of the *P. expansum* isolates exhibited reduced sensitivity to Dif, suggesting a notable decrease in the fungicide’s efficacy in controlling the most prevalent post-harvest apple pathogen. Moreover, Dif has demonstrated considerable efficacy in controlling various ascomycetes, including *Phacidiopycnis* spp., *Colletotrichum* spp., and *Alternaria* spp. [1,28,29].

On the other hand, the results showed a proportional increase in the inhibition percentages of *P. expansum* spore germination with the rising fungicide concentration. Complete inhibition began at a concentration of 0.5 µg/mL for some *P. expansum* isolates. At 1 µg/mL, Dif prevented the germination of over half of the tested isolates, while spore germination at 5 µg/mL was observed in only 9 isolates, with total germination inhibition at 10 µg/mL. In light of these findings, it appears that *P. expansum* sensitivity to Dif for spore germination was significantly higher than for mycelial growth, suggesting theoretically the use of Dif as a preventive rather than curative treatment. However, in practice, it is often challenging to determine when the inoculum can infect apples (before harvest, during harvest-related manipulations, or storage in warehouses). Therefore, effective treatment must have the potential to be applied both preventively and curatively [30,31,32].

The results of our study indicate a remarkable variability in the EC50 values for mycelial growth and spore germination of 100 isolates of *P. expansum*. Regarding mycelial growth, the EC50 values ranged from 0.027 to 1.673 µg/mL, with a mean of 0.263 µg/mL and a variation factor of 62.507. For spore germination, the EC50 values ranged from 0.0002 to 0.787 µg/mL, with a mean of 0.048 µg/mL and a very high variation factor of approximately 4113.835. Comparatively, the study conducted by Ali and Amiri [1] focused on 130 isolates of *P. expansum* and revealed mean EC50 values of 0.18 µg/mL for mycelial growth and 0.32 µg/mL for spore germination. The EC50 values ranged from 0.13 to 0.29 µg/mL for mycelial growth and from 0.19 to 0.37 µg/mL for spore germination inhibition, with respective variation factors of 2.23 and 1.95. Furthermore, the study carried out by Jurick et al. [10] examined 80 isolates of *P. expansum* and revealed a mean EC50 value of 0.14 µg/mL for mycelial growth. The EC50 values for mycelial growth ranged between 0.040 and 0.827 µg/mL, with a variation factor of 20.67. From this comparison, it appears that the mean EC50 for mycelial growth in the isolates of our study was higher compared to that of the two referenced studies, which could indicate a reduced effectiveness of Dif against the mycelial growth of the isolates studied. However, the opposite was inferred for spore germination. Additionally, the exceptionally high variation factors may reflect a diversity in the responses of *P. expansum* isolates to Dif, likely stemming from the variability in sampling over time and space.

The distribution of EC50 values for mycelial growth and spore germination across different intervals further underscored the variability in the sensitivity of *P. expansum* isolates to Dif. The presence of certain isolates, even at high EC50 concentrations, demonstrated a tolerance of *P. expansum* to Dif. In this context, a previous study on the sensitivity of *P. expansum* to tebuconazole, belonging to the same chemical family as Dif (DMI), also reported an extensive distribution of EC50 values across multiple intervals, indicating reduced sensitivity to this fungicide [8]. It is worth noting that five isolates of *P. expansum* exhibited an effective concentration to inhibit half of the mycelial growth greater than 0.920 µg/mL. Based on a previous study, Dif-resistant *P. expansum* isolates had an EC50 between 0.92 and 1.4 µg/mL [1]; therefore, five isolates from our study could be Dif-resistant. These isolates of *P. expansum* resistant to difenoconazole are primarily concentrated in the Meknes region. This situation can be explained by the frequent use of this fungicide for post-harvest treatments of apples in this area, especially after the withdrawal of several active substances such as thiophanate-methyl and carbendazim from the list of authorized plant protection products for controlling post-harvest diseases. This finding is supported by the study conducted by Khadiri et al. [14], which found that the majority of treatments used prior to apple storage in the conservation stations of the Meknes region are based on difenoconazole (commercial product Score). Therefore, the repeated use of the same fungicide contributes to the emergence of resistant strains. Considering that Dif is an active substance belonging to the demethylation inhibitors family, acting by inhibiting a specific enzyme known as 14-α-demethylase, encoded by the fungus’s CYP51 gene. This enzyme is indispensable in the sterol biosynthesis process [11]. The main mechanisms of resistance to DMIs involve mutations in the Cyp51 gene encoding for 14α-demethylase, such as I309T, E297K, I330T, P384S, Glu169, E170, I387 M, L144F, and Y464S [33,34,35,36], or an overexpression of the Cyp51 gene [37]. According to the findings of the study conducted by Ali and Amiri [1], repeated use of Dif may lead to the emergence of resistance in *P. expansum*. The analysis of the complete sequences of the CYP51 gene, performed on wild and resistant isolates, revealed a mutation in codon 126, where tyrosine was replaced by phenylalanine (Y126F).

Several studies have demonstrated the emergence of resistant strains of *P. expansum* to other families of fungicides. For instance, Errampalli et al. [38] reported the development of resistance of *P. expansum* to Thiabendazole, which belongs to the benzimidazole class. Additionally, a study conducted in the State of Washington documented the occurrence of resistance to pyrimethanil (anilinopyrimidines) in *P. expansum* strains [39]. Similarly, authors have reported resistance to boscalid in the succinate dehydrogenase B (SdhB) subunit of the respiratory complex II of *P. expansum* [7]. Faced with the challenge of the emergence of resistant strains due to the repeated use of the same active ingredient with a single mode of action, the adoption of fungicides combining active ingredients with diverse modes of action may be effective in controlling *P. expansum*. Academy, containing Fludioxonil and Dif, serves as a good example illustrating the effectiveness of combining two active ingredients against *P. expansum* [10].

## 5. Conclusions

This study represents the first assessment of the sensitivity of *P. expansum* isolates to difenoconazole (Dif), a demethylation inhibitor (DMI), in Morocco. Our results reveal significant diversity in *P. expansum* responses to this fungicide. Nearly half of the tested isolates showed reduced sensitivity to Dif, and based on a reference study, five isolates could be considered resistant to this active ingredient. Notably, these resistant isolates are primarily concentrated in the Meknes region, a situation that can be attributed to the frequent use of this fungicide for post-harvest treatments of apples in the area, especially after the withdrawal of several active substances such as thiophanate-methyl and carbendazim from the list of authorized phytosanitary products for combating post-harvest diseases. Consequently, the repeated use of the same fungicide contributes to the emergence of resistant strains. In light of the increasing emergence of resistant pathogenic strains, the use of fungicides that combine multiple active ingredients targeting different sites of fungal action could be an effective solution among other strategies to control this post-harvest apple pathogen. These findings provide a solid foundation for the development of future apple fungal disease management programs during storage. However, in-depth research is needed to molecularly characterize difenoconazole-resistant isolates to better understand their resistance mechanisms to this DMI.

## Figures and Tables

**Table 1:** Data from *P. expansum* isolates tested for their sensitivity to difenoconazole.

Isolate	Year	Cultivar or Source	GPS Coordinates of Storage Stations	PAD
IT2	2020	SD	N 33°58′8.54976″; W 5°15′48.798″	Meknes
IT3	2020	GD	N 33°58′8.54976″; W 5°15′48.798″	Meknes
IT4	2020	GD	N 33°58′8.54976″; W 5°15′48.798″	Meknes
IT5	2020	GD	N 33°58′8.54976″; W 5°15′48.798″	Meknes
IT9	2020	GD	N 33°58′8.54976″; W 5°15′48.798″	Meknes
IT10	2020	GD	N 33°58′8.54976″; W 5°15′48.798″	Meknes
T5a	2020	GD	N 33°22′29.50392″; W 5°22′38.24184″	Ifrane
T10	2020	GD	N 33°22′29.50392″; W 5°22′38.24184″	Ifrane
T12b	2020	GD	N 33°22′29.50392″; W 5°22′38.24184″	Ifrane
M7a	2020	F	N 33°24′52.4106″; W 5°17′40.58628″	Ifrane
M11	2020	SD	N 33°24′52.4106″; W 5°17′40.58628″	Ifrane
MA2	2020	GD	N 33°25′31.72944″; W 5°14′37.7826″	Ifrane
MA5	2020	SD	N 33°25′31.72944″; W 5°14′37.7826″	Ifrane
MA6	2020	SD	N 33°25′31.72944″; W 5°14′37.7826″	Ifrane
A11	2020	SD	N 33°25′31.72944″; W 5°14′37.7826″	Ifrane
S2	2020	GD	N 33°21′49.25448″; W 5°22′5.33568″	Ifrane
S3	2020	GD	N 33°21′49.25448″; W 5°22′5.33568″	Ifrane
S5	2020	F	N 33°21′49.25448″; W 5°22′5.33568″	Ifrane
BNS2	2020	GD	N 33°53′54.5208″; W 5°26′47.76864″	Meknes
FMB8	2020	GD	N 33°41′10.21704″; W 5°30′7.88904″	El Hajeb
Bs2	2020	GD	N 33°49′37.4″; W 5°28′2.9″	Meknes
Bs3	2020	GD	N 33°49′37.4″; W 5°28′2.9″	Meknes
Bs(AS3)	2020	CRA	N 33°49′37.4″; W 5°28′2.9″	Meknes
Bs(AS7)	2020	CRA	N 33°49′37.4″; W 5°28′2.9″	Meknes
V5	2021	GD	N 33°45′35.1″; W 5°19′37.1″	El Hajeb
V6b	2021	GD	N 33°45′35.1″; W 5°19′37.1″	El Hajeb
V7	2021	GD	N 33°45′35.1″; W 5°19′37.1″	El Hajeb
V10	2021	GD	N 33°45′35.1″; W 5°19′37.1″	El Hajeb
Tg10	2021	GD	N 33°46′16.41″; W 5°20′42.0972″	El Hajeb
Tg11	2021	GD	N 33°46′16.41″; W 5°20′42.0972″	El Hajeb
Ag3	2021	GD	N 33°49′46.5456″; W 4°59′7.5876″	Sefrou
Ag6	2021	GD	N 33°49′46.5456″; W 4°59′7.5876″	Sefrou
Ag8	2021	GD	N 33°49′46.5456″; W 4°59′7.5876″	Sefrou
AO1	2021	GD	N 33°45′50.3568″; W 5°0′53.5644″	Sefrou
AO3	2021	GD	N 33°45′50.3568″; W 5°0′53.5644″	Sefrou
AO6	2021	GD	N 33°45′50.3568″; W 5°0′53.5644″	Sefrou
At1	2021	GD	N 33°46′28.4844″; W 5°1′20.3916″	Sefrou
At2	2021	GD	N 33°46′28.4844″; W 5°1′20.3916″	Sefrou
At3	2021	GD	N 33°46′28.4844″; W 5°1′20.3916″	Sefrou
CH2	2021	SD	N 33°42′42.588″; W 5°2′29.6484″	Sefrou
CH4	2021	GD	N 33°42′42.588″; W 5°2′29.6484″	Sefrou
Tl1	2021	GD	N 32°49′3.4032″; W 4°57′34.9056″	Midelt
Tl2	2021	GD	N 32°49′3.4032″; W 4°57′34.9056″	Midelt
Tl3	2021	GD	N 32°49′3.4032″; W 4°57′34.9056″	Midelt
Tl4	2021	GD	N 32°49′3.4032″; W 4°57′34.9056″	Midelt
Tl5	2021	GD	N 32°49′3.4032″; W 4°57′34.9056″	Midelt
Tl6	2021	GD	N 32°49′3.4032″; W 4°57′34.9056″	Midelt
TM1	2021	GD	N 32°59′49.1496″; W 4°52′24.3228″	Midelt
TM4	2021	DG	N 32°59′49.1496″; W 4°52′24.3228″	Midelt
TM6	2021	GD	N 32°59′49.1496″; W 4°52′24.3228″	Midelt
TM7	2021	GD	N 32°59′49.1496″; W 4°52′24.3228″	Midelt
Aby4	2021	GD	N 32°45′2.2896″; W 5°1′45.1776″	Midelt
Ml4	2021	GD	N 32°44′56.5944″; W 5°1′45.3864″	Midelt
HM1	2021	GD	N 32°54′7.4664″; W 4°58′12.5256″	Midelt
HM3a	2021	GD	N 32°54′7.4664″; W 4°58′12.5256″	Midelt
AH1	2021	GD	N 32°46′3.4356″; W 5°0′8.136″	Midelt
AH2	2021	GD	N 32°46′3.4356″; W 5°0′8.136″	Midelt
AH3	2021	GD	N 32°46′3.4356″; W 5°0′8.136″	Midelt
AH4	2021	GD	N 32°46′3.4356″; W 5°0′8.136″	Midelt
BK3	2021	GD	N 32°46′12.5688″; W 4°59′56.4684″	Midelt
BK5	2021	F	N 32°46′12.5688″; W 4°59′56.4684″	Midelt
BK7	2021	GD	N 32°46′12.5688″; W 4°59′56.4684″	Midelt
ASL1	2021	GD	N 32°43′16.4028″; W 5°3′13.3488″	Midelt
ASL4	2021	GD	N 32°43′16.4028″; W 5°3′13.3488″	Midelt
ASL6	2021	GD	N 32°43′16.4028″; W 5°3′13.3488″	Midelt
DA2	2021	DG	N 32°40′27.5052″; W 5°15′30.9204″	Midelt
DA3	2021	GD	N 32°40′27.5052″; W 5°15′30.9204″	Midelt
DA5	2021	GD	N 32°40′27.5052″; W 5°15′30.9204″	Midelt
DA7	2021	GD	N 32°40′27.5052″; W 5°15′30.9204″	Midelt
DA8	2021	F	N 32°40′27.5052″; W 5°15′30.9204″	Midelt
PR2	2021	SD	N 33°47′43.5336″; W 5°29′41.226″	Meknes
DN2	2021	SD	N 33°44′28.5324″; W 5°28′21.0756″	Meknes
DN3	2021	F	N 33°44′28.5324″; W 5°28′21.0756″	Meknes
DN9	2021	SD	N 33°44′28.5324″; W 5°28′21.0756″	Meknes
DN10	2021	GD	N 33°44′28.5324″; W 5°28′21.0756″	Meknes
AML8	2021	GD	N 33°48′28.6812″; W 5°22′35.2884″	El Hajeb
OS14	2021	GD	N 33°55′35.526″; W 4°54′1.9584″	Sefrou
OS15	2021	DG	N 33°55′35.526″; W 4°54′1.9584″	Sefrou
CB1	2021	GD	N 33°51′36.2484″; W 4°29′56.94″	Sefrou
ZA1	2021	GD	N 33°49′9.39″; W 4°45′58.9068″	Sefrou
ZA2	2021	GD	N 33°49′9.39″; W 4°45′58.9068″	Sefrou
ZA3	2021	GD	N 33°49′9.39″; W 4°45′58.9068″	Sefrou
DKS2b	2022	SD	N 33°49′39.2592″; W 5°31′8.1372″	Meknes
AM2	2022	GD	N 33°45′49.4352″; W 5°20′15.2448″	El Hajeb
LM1	2022	GD	N 33°46′38.1036″; W 5°22′54.2928″	El Hajeb
FN1	2022	GD	N 33°58′4.116″; W 5°14′15.8928″	Meknes
FN8	2022	GD	N 33°58′4.116″; W 5°14′15.8928″	Meknes
MY8	2022	GD	N 33°59′36.8088″; W 5°12′2.4948″	Meknes
MY10	2022	SD	N 33°59′36.8088″; W 5°12′2.4948″	Meknes
BNS3	2022	SD	N 33°53′54.5208″; W 5°26′47.76864″	Meknes
BNS4	2022	GD	N 33°53′54.5208″; W 5°26′47.76864″	Meknes
BNS5	2022	SD	N 33°53′54.5208″; W 5°26′47.76864″	Meknes
BNS8	2022	SD	N 33°53′54.5208″; W 5°26′47.76864″	Meknes
BNS25	2022	GD	N 33°53′54.5208″; W 5°26′47.76864″	Meknes
BNS32	2022	GD	N 33°53′54.5208″; W 5°26′47.76864″	Meknes
AML13	2022	GD	N 33°48′28.6812″; W 5°22′35.2884″	El Hajeb
AML17	2022	GD	N 33°48′28.6812″; W 5°22′35.2884″	El Hajeb
AML18	2022	GD	N 33°48′28.6812″; W 5°22′35.2884″	El Hajeb
AML25b	2022	GD	N 33°48′28.6812″; W 5°22′35.2884″	El Hajeb
TG3	2022	GD	N 33°46′16.41″; W 5°20′42.0972″	El Hajeb

Cultivars: SD: “Starking Delicious”; GD: “Golden Delicious”; F: “Fuji”; DG: “Dorsett Golden”. CRA: Cold Room Atmosphere. PAD: Provincial Agriculture Directorate.

**Table 2:** Percentage of Inhibition (PI, %) and Effective Concentration (EC50) of Difenoconazole for 50% Inhibition of Mycelial Growth (MG) and Spore Germination (SG) in 100 *P. expansum* Isolates.

Isolate	PAD	PI (MG)				PI (SG)				EC50 (MG) µg/mL	EC50 (SG) µg/mL
		0.01 µg/mL	0.5 µg/mL	1 µg/mL	5 µg/mL	0.01 µg/mL	0.5 µg/mL	1 µg/mL	5 µg/mL		
IT2	Meknes	18.25	59.10	69.96	100	33.96	100	100	100	0.158	0.027
IT3	Meknes	19.53	63.23	68.81	100	50.93	95.06	100	100	0.143	0.009
IT4	Meknes	21.18	62.52	71.94	100	47.95	86.30	100	100	0.130	0.013
IT5	Meknes	20.99	58.57	69.83	100	42.81	95.89	100	100	0.145	0.017
IT9	Meknes	16.87	60.21	67.17	78.35	49.31	95.14	100	100	0.208	0.011
IT10	Meknes	20.29	65.29	68.72	81.71	23.51	40.73	88.08	100	0.155	0.076
T5a	Ifrane	9.89	52.44	60.11	100	45.45	82.21	100	100	0.262	0.017
T10	Ifrane	19.63	68.12	77.45	100	57.25	93.89	100	100	0.114	0.005
T12b	Ifrane	10.47	67.97	78.22	83.52	33.33	94.10	100	100	0.178	0.030
M7a	Ifrane	18.79	49.85	56.67	78.29	32.21	54.70	74.83	91.95	0.895	0.095
M11	Ifrane	31.94	44.66	49.65	72.86	51.82	72.12	79.09	100	1.442	0.011
MA2	Ifrane	12.73	63.75	73.29	100	35.76	78.79	100	100	0.167	0.033
MA5	Ifrane	12.10	60.56	68.88	83.20	38.64	96.27	100	100	0.223	0.022
MA6	Ifrane	18.90	58.61	65.98	79.43	40.40	100	100	100	0.200	0.019
MA11	Ifrane	11.65	60.69	68.38	82.96	58.62	96.26	100	100	0.228	0.004
S2	Ifrane	14.09	70.56	73.88	100	56.89	94.70	100	100	0.141	0.005
S3	Ifrane	6.21	62.92	69.30	100	53.57	95.71	100	100	0.217	0.007
S5	Ifrane	10.97	54.51	64.26	75.48	43.66	96.83	100	100	0.301	0.016
BNS2	Meknes	15.09	62.67	69.67	100	22.22	81.25	88.89	100	0.166	0.068
FMB8	El Hajeb	14.05	57.25	65.39	82.22	50.00	95.33	100	100	0.238	0.010
Bs2	Meknes	9.71	62.96	67.25	100	61.72	95.70	100	100	0.202	0.003
Bs3	Meknes	13.50	40.38	51.30	79.25	43.94	96.21	100	100	0.894	0.016
Bs(AS3)	Meknes	16.52	37.97	49.79	71.50	63.75	95.83	100	100	0.994	0.002
Bs(AS7)	Meknes	11.08	40.30	47.17	69.94	60.63	96.56	100	100	1.673	0.003
V5	El Hajeb	18.26	68.27	73.73	100	64.52	95.56	100	100	0.127	0.002
V6b	El Hajeb	8.26	62.23	70.68	100	69.32	94.89	100	100	0.204	0.0006
V7	El Hajeb	26.10	63.10	70.65	83.62	58.87	75.81	82.66	100	0.121	0.004
V10	El Hajeb	19.05	69.83	81.06	100	56.06	90.91	100	100	0.107	0.006
Tg10	El Hajeb	8.82	65.62	72.54	100	44.74	94.41	100	100	0.185	0.015
Tg11	El Hajeb	4.50	54.89	63.59	100	17.65	94.49	100	100	0.277	0.056
Ag3	Sefrou	8.53	59.05	66.10	100	80.92	98.03	100	100	0.226	0.013
Ag6	Sefrou	9.24	73.42	79.19	100	27.27	96.59	100	100	0.147	0.038
Ag8	Sefrou	4.49	64.90	73.16	100	53.85	92.31	100	100	0.210	0.007
AO1	Sefrou	3.90	68.42	74.46	100	30.77	96.63	100	100	0.198	0.033
AO3	Sefrou	4.82	58.61	68.27	84.41	40.00	95.63	100	100	0.283	0.021
AO6	Sefrou	13.87	59.86	66.09	77.21	68.42	73.68	94.41	100	0.238	0.0002
At1	Sefrou	10.39	76.31	81.06	88.07	18.27	92.31	97.60	100	0.143	0.058
At2	Sefrou	26.72	81.71	86.43	100	61.67	100	100	100	0.054	0.003
At3	Sefrou	16.61	80.61	84.61	100	7.14	94.20	100	100	0.092	0.075
CH2	Sefrou	20.06	72.91	78.62	84.09	28.57	96.43	100	100	0.111	0.036
CH4	Sefrou	12.79	68.76	75.54	83.83	16.52	87.50	92.86	100	0.169	0.069
Tl1	Midelt	10.55	50.47	63.14	100	26.67	80.00	93.33	100	0.498	0.054
Tl2	Midelt	18.45	68.28	72.87	79.50	22.29	40.29	58.57	95.43	0.149	0.712
Tl3	Midelt	16.53	70.85	75.03	100	45.00	86.67	100	100	0.126	0.017
Tl4	Midelt	10.07	55.85	64.02	100	57.50	83.33	90.00	94.17	0.234	0.003
Tl5	Midelt	11.14	67.72	72.31	100	55.00	76.67	93.33	100	0.167	0.005
Tl6	Midelt	10.58	55.17	61.27	100	58.18	72.27	92.73	100	0.243	0.003
TM1	Midelt	4.48	69.01	73.58	82.98	23.21	69.64	91.96	100	0.227	0.076
TM4	Midelt	2.70	67.03	73.93	83.96	57.14	82.14	92.86	100	0.242	0.003
TM6	Midelt	3.01	72.76	77.20	100	55.42	93.33	100	100	0.184	0.004
TM7	Midelt	5.67	57.45	64.56	82.73	24.04	77.88	92.79	100	0.302	0.063
Aby4	Midelt	10.30	46.95	72.34	100	31.58	94.74	100	100	0.520	0.057
Ml4	Midelt	6.36	66.98	81.31	100	17.65	66.91	96.69	100	0.173	0.095
HM1	Midelt	10.45	40.87	74.00	100	16.67	88.89	94.44	100	0.563	0.077
HM3a	Midelt	9.28	51.60	69.62	100	6.67	86.67	96.25	100	0.358	0.088
AH1	Midelt	10.90	77.02	83.37	100	56.25	100	100	100	0.124	0.004
AH2	Midelt	4.49	74.34	81.21	100	29.03	96.77	100	100	0.163	0.035
AH3	Midelt	5.97	73.32	78.95	100	48.96	69.44	83.33	100	0.164	0.012
AH4	Midelt	6.48	70.59	76.36	100	32.41	85.86	94.48	100	0.174	0.036
BK3	Midelt	10.15	63.65	70.66	85.80	38.46	93.75	100	100	0.207	0.023
BK5	Midelt	9.96	64.51	72.67	100	18.75	69.64	76.79	100	0.178	0.116
BK7	Midelt	10.43	63.39	74.21	82.72	58.33	96.35	100	100	0.216	0.004
ASL1	Midelt	11.66	76.39	80.02	100	35.71	79.93	87.07	100	0.128	0.033
ASL4	Midelt	11.11	75.80	79.23	100	18.52	92.13	100	100	0.133	0.056
ASL6	Midelt	9.57	79.54	83.34	100	38.46	69.23	94.23	100	0.124	0.033
DA2	Midelt	20.99	63.22	67.98	85.96	50.96	95.67	100	100	0.153	0.009
DA3	Midelt	6.67	62.73	71.38	81.70	23.53	98.90	100	100	0.247	0.043
DA5	Midelt	8.88	62.02	69.71	84.03	36.96	99.01	100	100	0.235	0.024
DA7	Midelt	13.85	26.73	59.94	82.84	47.50	66.67	93.33	100	0.971	0.011
DA8	Midelt	3.70	60.09	70.66	85.32	47.06	93.75	100	100	0.272	0.013
PR2	Meknes	19.22	68.95	74.87	100	6.67	97.08	100	100	0.119	0.074
DN2	Meknes	15.59	66.62	71.13	81.36	40.23	78.43	91.25	100	0.175	0.025
DN3	Meknes	19.05	63.28	69.50	79.36	25.50	85.91	93.62	96.64	0.171	0.059
DN9	Meknes	2.42	26.13	38.68	71.82	15.38	33.17	61.54	100	1.582	0.787
DN10	Meknes	9.54	65.28	77.49	100	13.39	92.86	100	100	0.170	0.065
AML8	El Hajeb	20.94	69.28	76.51	100	73.33	96.25	100	100	0.108	0.0002
OS14	Sefrou	15.87	74.83	80.68	86.51	7.25	55.07	78.26	100	0.121	0.168
OS15	Sefrou	9.95	50.67	63.11	74.68	25.00	68.75	90.63	100	0.495	0.073
CB1	Sefrou	9.17	82.09	86.18	100	55.15	84.19	100	100	0.116	0.004
ZA1	Sefrou	15.27	100	100	100	36.08	94.94	100	100	0.050	0.026
ZA2	Sefrou	10.87	68.30	74.96	86.06	26.67	60.00	75.83	85.00	0.180	0.116
ZA3	Sefrou	12.33	35.67	73.89	100	48.57	77.14	82.86	88.57	0.684	0.012
DKS2b	Meknes	20.43	68.51	74.88	100	54.23	90.91	100	100	0.114	0.007
AM2	El Hajeb	13.80	65.34	76.06	100	51.98	89.83	96.89	100	0.151	0.008
LM1	El Hajeb	42.95	71.75	81.04	100	49.82	90.88	96.49	100	0.027	0.010
FN1	Meknes	11.48	66.92	69.02	80.64	39.94	77.67	97.48	100	0.207	0.029
FN8	Meknes	13.99	75.89	79.28	86.35	22.49	71.01	93.20	100	0.131	0.076
MY8	Meknes	14.73	61.14	67.60	82.96	25.42	63.05	88.14	100	0.207	0.102
MY10	Meknes	15.23	75.73	78.74	84.14	23.36	81.58	94.41	100	0.128	0.057
BNS3	Meknes	18.45	64.33	72.48	84.31	31.82	87.34	100	100	0.156	0.037
BNS4	Meknes	24.49	61.97	69.39	83.02	43.73	92.28	100	100	0.139	0.017
BNS5	Meknes	22.03	71.43	81.00	100	50.32	82.28	93.67	95.89	0.092	0.010
BNS8	Meknes	19.20	69.03	77.22	85.45	21.82	75.57	84.04	100	0.127	0.081
BNS25	Meknes	18.02	64.65	72.33	77.06	18.69	93.77	97.38	100	0.170	0.056
BNS32	Meknes	14.74	65.67	73.75	82.03	37.17	96.17	100	100	0.177	0.024
AML13	El Hajeb	27.04	60.23	70.67	100	26.02	82.45	95.30	97.81	0.110	0.062
AML17	El Hajeb	19.45	62.96	77.43	82.36	23.05	90.91	100	100	0.147	0.049
AML18	El Hajeb	23.22	72.19	81.46	100	19.75	85.03	100	100	0.087	0.059
AML25b	El Hajeb	19.00	63.92	74.58	100	44.05	76.79	88.99	95.83	0.132	0.018
TG3	El Hajeb	10.81	69.92	79.17	100	44.41	91.78	96.38	100	0.148	0.016

PAD: Provincial Agriculture Directorate. Each value represents the average of four replicates. The concentration of 10 µg/mL of difenoconazole completely inhibited (PI = 100%) mycelial growth and spore germination of all *P. expansum* isolates.

**Table 3:** Analysis of the effective concentration of difenoconazole required to inhibit 50% of mycelial growth and spore germination (EC50) for all *P. expansum* isolates (*n* = 100), compared to a previous study.

EC50 (µg/mL)	Mycelial Growth	Spore Germination	Reference
Mean	0.263	0.048	This study
Min	0.027	0.0002	
Max	1.673	0.787	
VF *	62.507	4113.835	
Mean	0.18	0.32	[1]
Min	0.13	0.19	
Max	0.29	0.37	
VF *	2.23	1.95	

* VF: Variation factor.

## Data Availability

Data is contained within the article.

## Supplementary Materials

The following supporting information can be downloaded at: https://www.mdpi.com/article/10.3390/microorganisms12112169/s1, Figure S1. Chemical structure of difenoconazole (A) and thiabendazole (B), [40,41]; Figure S2. Symptomatic apple sampled from the cold storage rooms of storage facilities in Morocco; Table S1. The variance analysis of the fungicide concentration on the inhibition percentage of different isolates.
