# Supplementary material for: In Vitro Assessment of Penicillium expansum Sensitivity to Difenoconazole
Source: Microorganisms. 2024 Oct 28;12(11):2169. doi: 10.3390/microorganisms12112169 (PMC11596040; doi:10.3390/microorganisms12112169)
Supplement: Supplementary file 1 [file microorganisms-12-02169-s001.zip › microorganisms-3259941-supplementary.pdf]

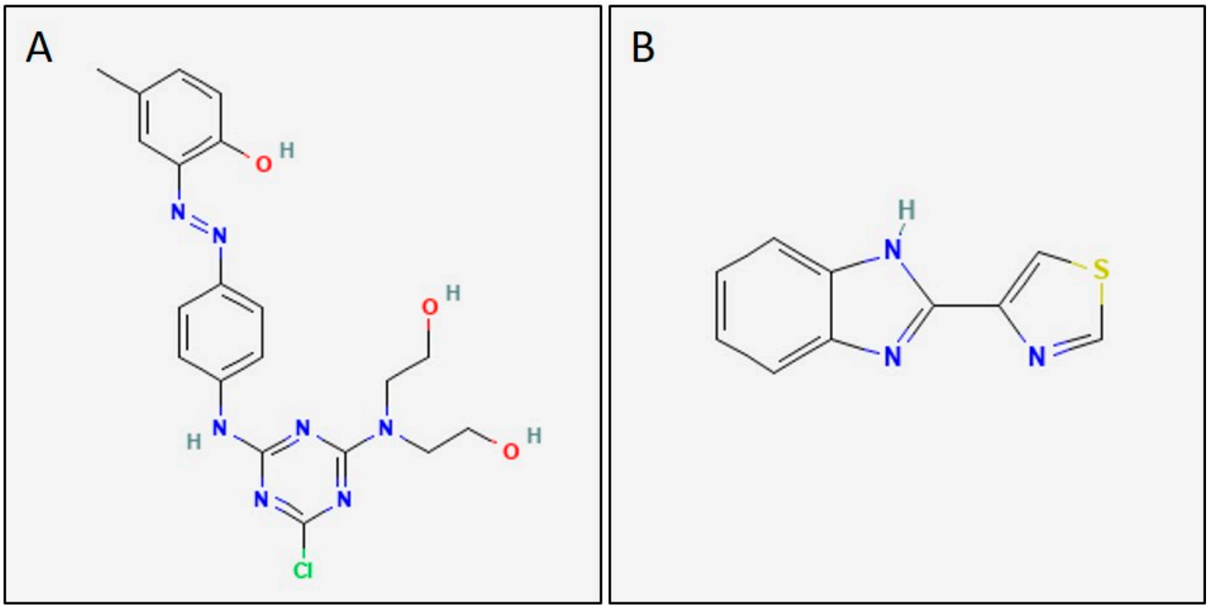

Figure S1. Chemical structure of difenoconazole (A) and thiabendazole (B) [40,41]

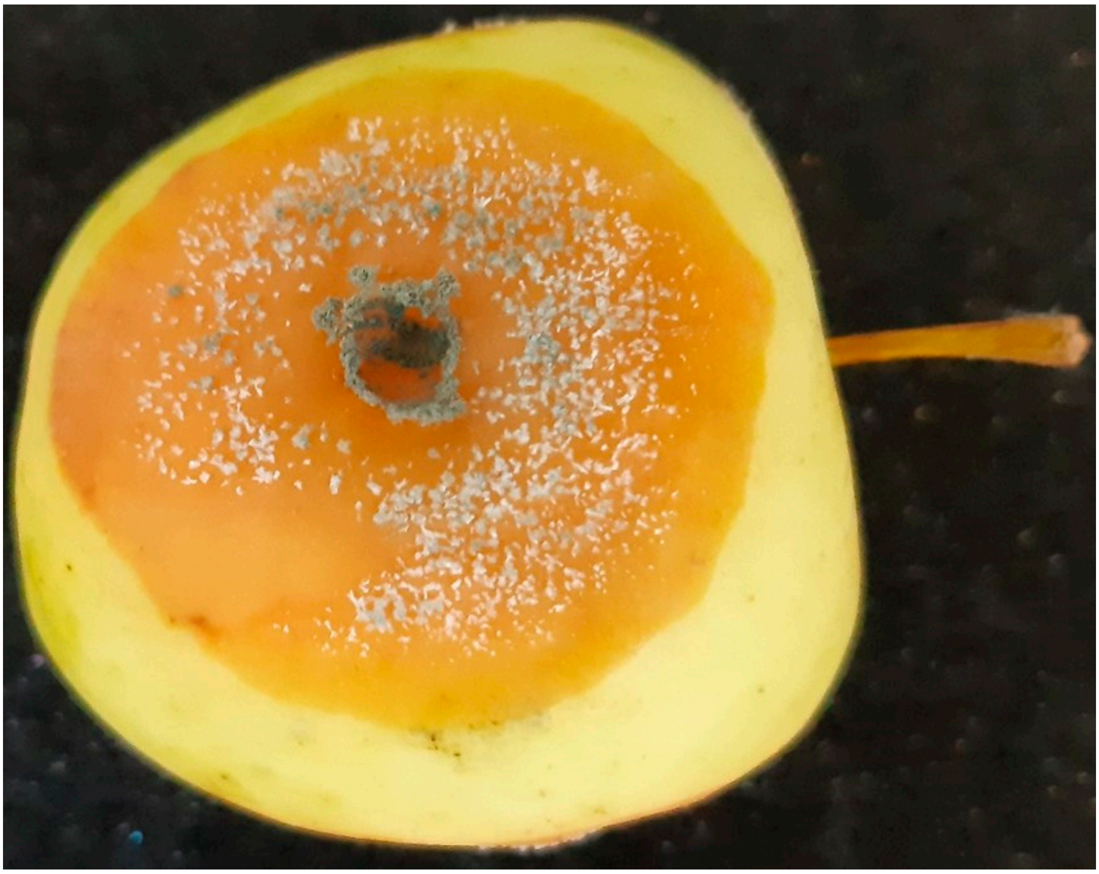

Figure S2. Symptomatic apple sampled from the cold storage rooms of storage facilities in Morocco

Table S1. The variance analysis of the fungicide concentration on the inhibition percentage of different isolates

Bioassay	Concentration		Isolate		Concentration * Isolate	
	F	P	F	P	F	P
Mycelial growth	123292.753	0.000	179.922	0.000	82.567	0.000
Spore germination	112894.106	0.000	376.497	0.000	119.551	0.000

[40] S. Rocchi, N. Morin-Crini, C. Léchenault-Bergerot, C. Godeau, M. Fourmentin, L. Millon, G. Crini, Effet de l'interaction cyclodextrine-difénoconazole sur la croissance d'une moisissure responsable d'infections fongiques graves, Environnement, Risques & Santé. 18 (2019) 411–417.

[41] D.B. SORO, D.L. KOUADIO, K.N. ABOUA, M. DIARRA, L. MEITE, K.S. TRAORE, Dégradation photocatalytique du thiabendazole en solution aqueuse, Afrique Sci. 14 (2018) 55–63.
